# Green and white MEKC for determination of different anti-diabetic binary mixtures and their triple-combo pill

**DOI:** 10.1186/s13065-023-00997-0

**Published:** 2023-07-25

**Authors:** Aya R. Ahmed, Mohamed A. Korany, Shereen M. Galal, Marwa A. A. Ragab

**Affiliations:** Department of Pharmaceutical Analytical Chemistry, Faculty of Pharmacy, Alexandria University, El-Messalah, Alexandria, 21521 Egypt

**Keywords:** Empagliflozin, Greenness and whiteness appraisal, Linagliptin, Metformin, Micellar electrokinetic chromatography

## Abstract

The work introduces green and white sustainable micellar electrokinetic chromatography (MEKC) procedure that could analyze therapeutically related drugs, empagliflozin (EMP), linagliptin (LIN) and metformin (MET) which are antidiabetic drugs with different mechanism of action, in their different pharmaceutical combinations. The method not only comply with the green analytical concepts, but also it is in line with sustainable analytical concepts as it is economic by applying the same operating conditions to analyze different pharmaceuticals in quality control (QC) labs which is a crucial step in QC labs and research centers to save time, effort, and money. Moreover, the method functionality regarding its scope with its achieved levels of accuracy, precision, low detection, and quantitation limits is tested using white assessment tool and compared with reported methods. The proposed MEKC coupled with a diode array detector (DAD) has been developed and validated for micro estimation of EMP and LIN in their low critical concentrations with MET in a ratio of (EMP: MET, 1:40) and (LIN: MET, 1:200). Separation was achieved within 6 min using fused silica capillary (40 cm × 50 µm id) using 20 mM Tris buffer (pH 10) in presence of 50 mM sodium dodecyl sulphate and 10% v/v methanol. The concentration ranges of the studied anti-diabetic drugs were 10–500, 10–100 and 2.5–100 µg. mL^−1^ for MET, EMP and LIN, respectively. The developed method is the first MEKC for concurrent determination of EMP, LIN and MET with high separation efficiency, low solvent consumption and regard as an easy green and white analytical tool. Moreover, Greenness and whiteness assessment were done via the most widely used Analytical Eco-Scale, the innovative AGREE tool and the RGB 12 algorithm.

**Supplementary Information:**

The online version contains supplementary material available at 10.1186/s13065-023-00997-0.

## Introduction

The greenness and whiteness of analytical methodology is vital and significant with the aim of improving the health hazards and environmental effect. It is important not only to comply with the green analytical concepts, but also should be in line with sustainable analytical concepts [1].

A novel triple combo pill (Trijardy XR^®^) has been accepted officially by FDA (January 2020) which merged into one fixed dose tablet three classes of commonly used anti-diabetic medications working together to reduce blood glucose level in type 2 diabetes mellitus(T2DM). These drugs are characterized by diverse mechanism of action, with both short and long-term glycemic control, achieving low hypoglycemic risk in order to accomplish effectiveness and tolerability for different individuals [2].

**Fig. 1:**
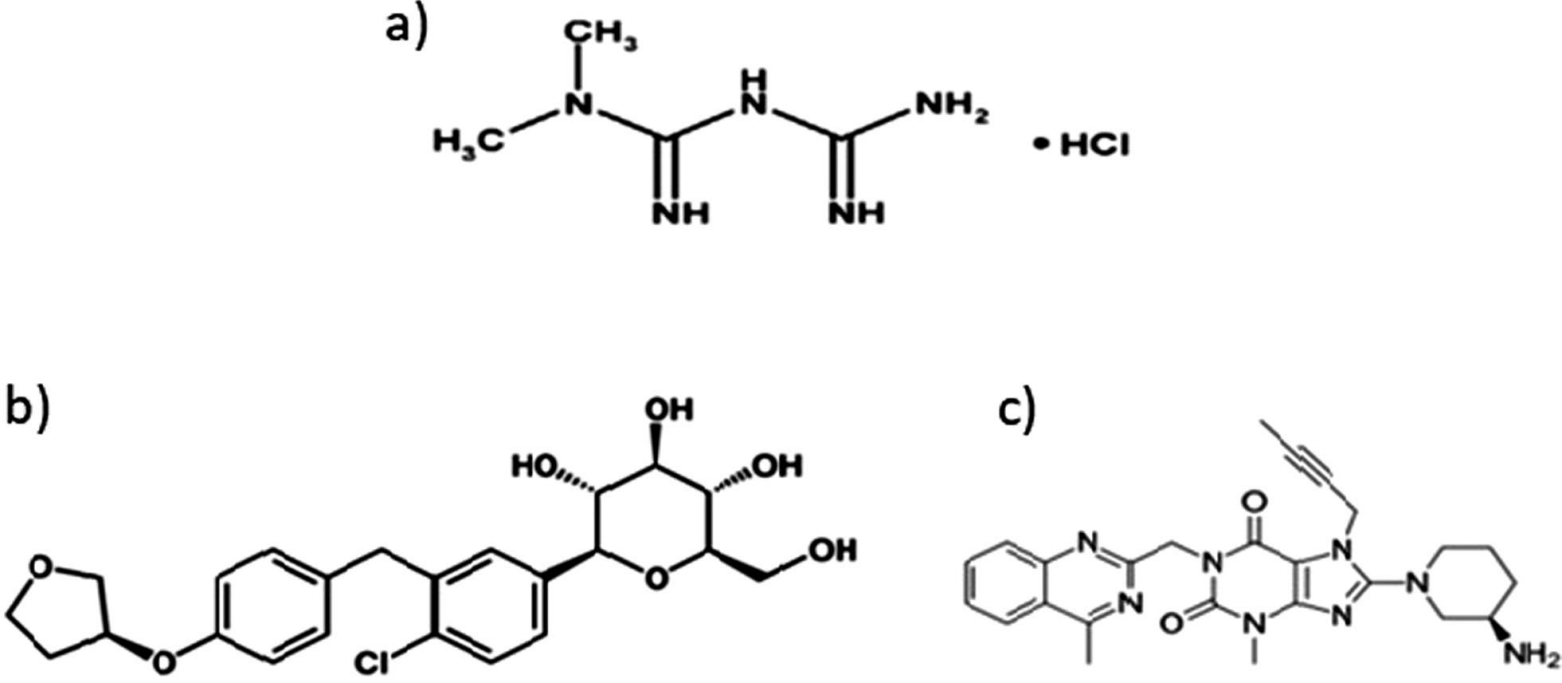
Chemical structure of **a** Metformin HCl **b** Empagliflozin **c** Linagliptin

Metformin (MET) Fig. 1a, acts as an insulin sensitizer mainly recommended for patients with T2DM [3], while Empagliflozin (EMP) Fig. 1b, enables the elimination of glucose in urine [4]. Linagliptin (LIN) Fig. 1c, is used in treatment of T2DM as it regulates glucose homeostasis [5].

Literature review showed that limited methods of analysis were established for simultaneous determination of the triple combination such as HPLC [6–8], HPTLC[6, 9], UPLC [10] and spectrophotometry [11],whereas, for their binary combinations, HPLC [12–14], HPTLC [15–17], UPLC [17], voltammetric method [18] and spectrophotometric methods were reported [19–21].

To our knowledge, MEKC method has not been applied yet for the determination of the combo pill triple combination therapy and their related binary combinations of EMP/MET or LIN/EMP except for LIN/MET mixture, one CE method was found in literature [22].

The current work represents green and white MEKC procedure for analyzing the three therapeutically related anti-diabetic drugs (EMP/LIN/MET) in their different binary and ternary pharmaceutical combinations. The method not only comply with the green analytical concepts, but also it is in line with sustainable analytical concepts as it is economic by applying one analytical technique with one operating condition which saves time, money and efforts in QC laboratories. Moreover, the two green chemistry metrics along with the white RGB 12 model revealed that our proposed MEKC is the most green and white compared to other published methods.

## Methods

### Instrumentation

Agilent CE instruments 7100 series from Agilent Technologies, Waldbronn, Germany, equipped with a DAD was used. It was also equipped with a data handling system comprising a computer loaded with software “Agilent Openlab CDS ChemStation”. The used capillary obtained from Agilent Technologies, Inc. It was a deactivated fused silica capillary that had the following dimensions: 48.5 cm total length, 40 cm effective length, and 50 µm id. For the purpose of accurate weighing of buffer and surfactant, a 3-digits Sartorius BL 310 balance was used, while the analytical balance used for accurate weighing of the drugs and the dosage forms a 4-digit Kern AEJ 220-4 M balance (Germany) was used. In addition, a Jenway 3305 pH Meter was used for pH measurements.

### Materials and reagents

Pharco Pharmaceuticals, Co. (Alexandria, Egypt) kindly offered MET and LIN, while EMP was supplied by Pharaonia pharmaceuticals (Alexandria, Egypt). HPLC grade methanol (Fisher Scientific UK Limited, Loughborough, Leicestershire, UK), Tris buffer Extra pure and sodium phosphate dibasic (Loba Chemie, Mumbai, India), sodium hydroxide, hydrochloric acid, sodium dodecyl sulphate (El-Nasr chemical Co., Giza, Egypt) and deionized water were used.

Empagliform® tablets labeled to contain 12.5 mg EMP/ 500 mg MET, Gliptalina® tablets labeled to contain 2.5 mg LIN/ 500 mg MET and Glyxambi® tablets labeled to contain 10 mg EMP/ 5 mg LIN were purchased from the Egyptian market. Trijardy XR® tablets labeled to contain 25 mg EMP/ 5 mg LIN/ 1000 mg MET, due to the unavailability in the local market, laboratory-prepared tablets having the same drugs’ amount were prepared. This was done by using the individual dosage forms of each drug alone Trajenta®, Empaglimax^®^ and Cidophage^®^ labelled to contain 5 mg, 25 mg and 1000 mg per tablet, respectively.

### Conditions of MEKC separation

#### Preparation of running buffer

Tris buffer (20 mM, pH 10) was prepared by dissolving 0.24 g of Tris buffer extra pure in a 100-mL volumetric flask using deionized water, pH was adjusted to 10 with 0.5 M hydrochloric acid. To prepare 50 mM of sodium dodecyl sulphate (SDS) in Tris buffer, the following was done: a weight of 1.44 g SDS was added to the prepared buffer, followed by 15 min sonication till reach the complete dissolution. The finally used background electrolyte (BGE) is 20 mM Tris buffer adjusted to pH 10 with 0.5 M hydrochloric acid. This buffer contains 50 mM sodium dodecyl sulphate (SDS) and 10%v/v methanol.

#### MEKC-DAD procedure

The fused silica capillary (40 cm × 50 µm id) was conditioned at the start of each working day first for 10 min using 0.5 M NaOH followed by another 10 min with deionized water, then for 5 min using NaOH (0.1 M), again deionized water was utilized for 5 min and the last 10 min of conditioning Tris buffer solution was used. For the in-between injections, two min flushing was done using the selected Tris buffer. The capillary was kept at 25 °C. The BGE used was a 20 mM Tris buffer (pH 10) contains 50 mM SDS and 10%v/v methanol, prepared as described under Sect. "Preparation of running buffer:". Into the auto sampler, these prepared samples were placed and hydrodynamic injection was done at the anodic side (50 mbar pressure for 15 s). To quantify EMP, LIN and MET, a constant voltage (+ 30 kV) was applied during the analysis and the detections were performed at 230 nm.

### Preparation of stock and construction of the calibration graphs

Stock solutions 5000 µg. mL^−1^ of MET and 1000 µg. mL^−1^ of both EMP and LIN were prepared in methanol. These stock solutions were stable for at least one month when kept refrigerated at 4 ºC. Suitable aliquots from EMP, LIN and MET stock solutions were transferred separately into a series of 10-mL volumetric flasks in order to prepare their working solutions. This was followed by completing the volume using deionized water to reach the concentration ranges 10–500, 10–100 and 2.5–100 µg. mL^−1^ for MET, EMP and LIN, respectively.

### Analysis of pharmaceutical formulations

A number of ten tablets were weighed and finely powdered. The content of one powdered tablet equivalent to 25 mg EMP/5 mg LIN/1000 mg MET (Trijardy XR®, Mix I), 12.5 mg EMP/500 mg MET (Empagliform®, Mix II), 2.5 mg LIN/500 mg MET (Gliptalina^®^, Mix III), or 5 mg LIN/ 10 mg EMP( Glyxambi^®^,Mix IV) was extracted with 25 mL methanol in separate 25-mL volumetric flasks. Following sonication for 20 min and further filtration (Whatman No. 1 filter paper 110 mm diameter), volumes of 0.125 mL (Mix I), 0.25 mL (Mix II), 0.25 mL (Mix III) and 1.25 mL (Mix IV) were diluted with deionized water in separate 10-mL volumetric flasks to get working tablet solutions equivalent to 12.5 EMP/2.5 LIN/500 MET (Mix I), 12.5 EMP/500 MET (Mix II), 2.5 LIN/500 MET (Mix III) / 25 LIN and 50 EMP (Mix IV), respectively. Final concentrations of compounds in µg. mL^−1^.

These final solutions, representing each mixture from I to IV, were then analyzed using the proposed MEKC as explained in "MEKC-DAD procedure" section.

## Results and discussion

### Method optimization

First, capillary zone electrophoresis (CZE) technique at various pH range (3–10) using phosphate buffer was tried but it failed to analyze the triple combination due to pka value of EMP (pka,12.57), it is considered as a weakly ionizable compound [23]. It is not ionized in the common pH range used in CZE; the simplest technique used in CE. Consequently, the weakly ionizable EMP will be eluted by the electro-osmotic flow (EOF) causing poor peak shape.

As a result, MEKC was performed by adding a surfactant such as the anionic SDS exceeding its critical micelle concentration (CMC) to Tris buffer solution. In this mode, both neutral and charged molecules can be separated so it improved the peak shape and symmetry of EMP [24].The partitioning of the antidiabetic drugs will vary depending on its hydrophobic interaction with the micelle. Differences in the time that MET, EMP and LIN spent in the micellar phase will determine their separation [25]. MET spent less time in the micellar phase due to its poor lipophilicity compared to EMP and LIN [26]. On the other hand, LIN and EMP showed nearly the same hydrophobic nature, having the same Log P values equal to 1.7. So, their peaks appeared at the same migration time in each trial and by the aid of DAD both drugs proved to be co-eluted together.

Consequently, to solve this problem a modification was performed by the addition of organic modifier such as methanol to the BGE. Addition of methanol causes alterations in the viscosity and dielectric constant of the electrolyte, thus a significant reduction in the velocity of BGE and separation of the coeluted drugs EMP and LIN with reasonable resolution and migration time [27, 28].

The following parameters were studied; buffer type and pH, buffer strength, SDS concentration, percentage of organic modifier, applied voltage, applied pressure, injection time, operating temperature, and detection wavelength.

Two buffer systems (20 mM) were investigated namely, phosphate buffer and Tris buffer. In all the analytical conditions, phosphate buffer, on the contrary of tris buffer, was associated with high currents (40–65 µA), which lead to excessive heating of the capillary and their breakage [24, 29]. As a result, Tris was chosen to continue further studies for method optimization as no elevated current is generated into the instrument (20 µA).

The role of the buffer pH is crucial relative to the degree of ionization of drugs. The pKa values are: 12.57, 12.4 and 8.6 for EMP, MET and LIN respectively[30]. Consequently, a wide pH range (3,6,7,9 and 10) was tested using Tris for its effect on the MEKC behavior of the tested compounds in presence of 50 mM SDS and 10% v/v MeOH. MET and LIN peaks were well resolved with good symmetry and peak shape at all the studied pH range. On the other hand, no peak appears for EMP at the acidic pH 3 while at higher pH from 6 to 9, EMP had distorted peak shape or poor symmetry [31] (Additional file 1: Fig. S1). Finally, Tris buffer at pH 10.0 was chosen to continue the current study and was suitable for further method optimization.

Tris buffer solutions (pH 10.0) at different strength values (10, 20 and 40 mM) were tested for its effect on migration time and peak shape. Low buffer strength decreased the resolution between EMP and LIN with value 1.4 (< 2), while upon increasing the strength of Tris buffer, it insignificantly increased the migration time of the EMP, LIN and MET. A strength of 20 mM was optimum regarding the cited drugs peaks symmetry and reasonable migration times.

The effect of SDS concentration on EMP, LIN and MET separation was studied by addition of 25, 50 and 100 mM SDS in presence of 10% v/v methanol in the BGE. As SDS concentration increased, the migration time slightly increased as well. Despite the short migration time upon using 25 mM SDS, bad resolution was observed between EMP and LIN peaks. Therefore, 50 mM of SDS was selected for separation of MET, EMP and LIN as it is the best compromise between peaks shape and analysis time taking into consideration that they are well separated at the studied SDS concentrations (Additional file 1: Fig. S2a).

The amount of methanol had great effect on separation of the anti-diabetic compounds. EMP and LIN were co-eluted upon using methanol < 10% (v/v). Upon using 15% methanol, migration time of the compounds increased. The high content of methanol > 20% can hinder micelle formation [28]. Therefore, a concentration of 10% (v/v) of methanol as an organic modifier was selected as the optimum (Additional file 1: Fig. S2b).

The absorption spectra of EMP, LIN and MET, Additional file 1: Fig. S3, revealed that a wavelength of 230 nm showed high responses for the three drugs with low background and inactive ingredients interference, consequently it was the best in terms of high selectivity and sensitivity to be used for their simultaneous analysis. Moreover, wavelength 230 nm showed nearly maximum responses for estimation of EMP and LIN in their low critical concentrations with MET in their dosage forms ratio of (EMP: MET, 1:40) and (LIN: MET, 1:200).

Instrumental parameters optimization including applied voltage, applied pressure, injection time and operating temperature are discussed in the supplementary material file.

**Fig. 2:**
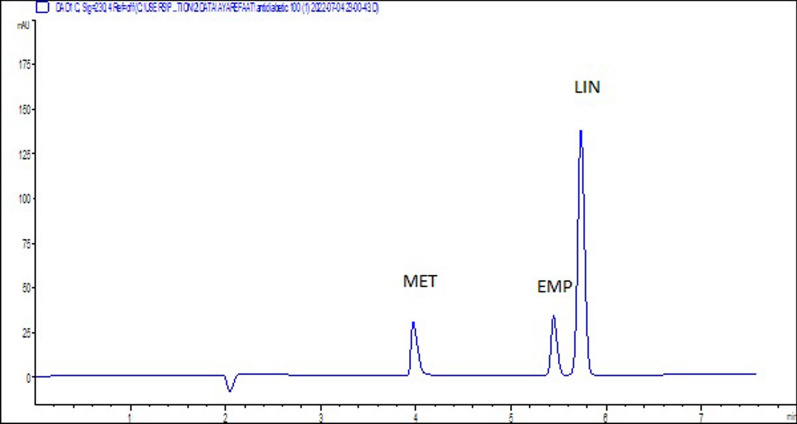
Typical electropherogram of a standard mixture of 100 µg. mL^−1^MET, EMP, and LIN detected at 230 nm

Final separation was achieved within 6 min using a BGE of Tris buffer (20 mM, pH 10.0) containing 50 mM of the anionic SDS and 10% v/v MeOH as an organic modifier at 25 ºC as an operating temperature, 30 kV as the applied voltage, 50 mbar and 15 s as pressure and injection time, respectively. The three anti-diabetic compounds (EMP, LIN and MET) were detected at wavelength 230 nm. A typical electropherogram of MET, EMP, and LIN, analyzed using the proposed MEKC conditions was shown in Fig. 2. Additional file 1: Table S1. summarized the system suitability parameters of the three antidiabetic compounds. Moreover, the peaks of EMP, LIN and MET were sharp, well-resolved, symmetric and eluted within reasonable migration time (tm) [32].

### Method validation

The proposed MEKC technique was fully validated as per the ICH guidelines [33].

#### Linearity

Linearity was assessed in the concentration ranges of 10–100 (EMP), 2.5–100 (LIN), and 10–500 (MET) µg. mL^−1^. Regression and statistical parameters were calculated as shown in Table 1. High degree of linearity indicated by high r values > 0.9992 and F values > 5404 with low Sy/x < 9.51 and significant F values (< 5.30 × 10^–8^) [34–37].

**Table 1:** Analytical parameters for determination of MET, EMP and LIN ternary mixture using the proposed MEKC-DAD method

Parameters	MET	EMP	LIN
Selected wavelength λ (nm)	230		
Concentration range (µg. mL^−1^)	10–500	10–100	2.5–100
Regression equation: Intercept (a) Slope (b)	−4.41 1.98	−1.99 1.40	−2.70 7.96
Correlation coefficient (r)	0.9996	0.9997	0.9992
S_a_^a^	5.33	0.60	6.20
S_b_^b^	0.02	0.01	0.11
S_y/x_^c^	8.55	0.89	9.51
RSD% of the slope (S_b_%)	1.01	0.71	1.38
F^d^	10,635.62	17,987.06	5404.57
Significance F	5.30 × 10^–8^	1.85 × 10^–8^	2.05 × 10^–8^
LOD^e^ (µg. mL^−1^)	3	3	1
LOQ^f^ (µg. mL^−1^)	10	10	2.5

^a^Standard deviation of the intercept

^b^Standard deviation of the slope

^c^Standard deviation of the residuals

^d^Variance ratio, equals the mean of squares due to regression divided by the mean of squares about regression (due to residuals)

^e^LOD = Limit of detection

^f^LOQ = Limit of quantitation

#### Limits of detection (LOD) and of quantitation (LOQ)

LOD and LOQ of EMP, LIN and MET were determined based on S/N ratio of 3: 1 for LOD and 10: 1 for LOQ. The proposed MEKC method yielded low LOD (3, 1 and 3 µg. mL^−1^) and LOQ (10, 2.5 and 10 µg. mL^−1^) values for EMP, LIN and MET, respectively.

#### Accuracy and precision

Method accuracy and precision were validated by analyzing three synthetic mixtures with different concentrations of EMP, LIN and MET. Referring to Additional file 1: table S2, error values (< 1.97) indicated high level of method accuracy and low values of RSD% less than 2 pointing out acceptable precision.

The intra-day precision (RSD%) ranged from 0.39 to 1.66%, 0.25 to 1.84% and 0.47 to 1.98% for EMP, LIN and MET, respectively. Inter-day precision ranged from 0.42 to 0.66%, 0.91 to 1.78% and 0.57 to 1.96% for EMP, LIN and MET, respectively.

Moreover, the proposed method could analyze accurately and precisely EMP and LIN in their low critical concentrations with MET (EMP: MET, 1:40) and (LIN: MET, 1:200).

#### Robustness

Various parameters in the proposed MEKC method were slightly changed such as: pH of buffer (9.8, 10.0, 10.2), buffer strength (18, 20, 22 mM), SDS concentration (48,50,52 mM), percent of organic modifier (9,10,11% V/V) in addition to detection wavelength (228, 230, 232 nm) were varied.

The impact of these minor changes on the migration time (tm) values and peaks area were considered by calculating the RSD% of the peak areas and SD of the migration times for each compound. The low values of RSD% (< 1.62) along with almost unchanged migration times values obtained after inducing minor intentional changes in the methods parameters revealing high degree of method’s robustness. (Additional file 1: Table S3).

#### Specificity

**Fig. 3:**
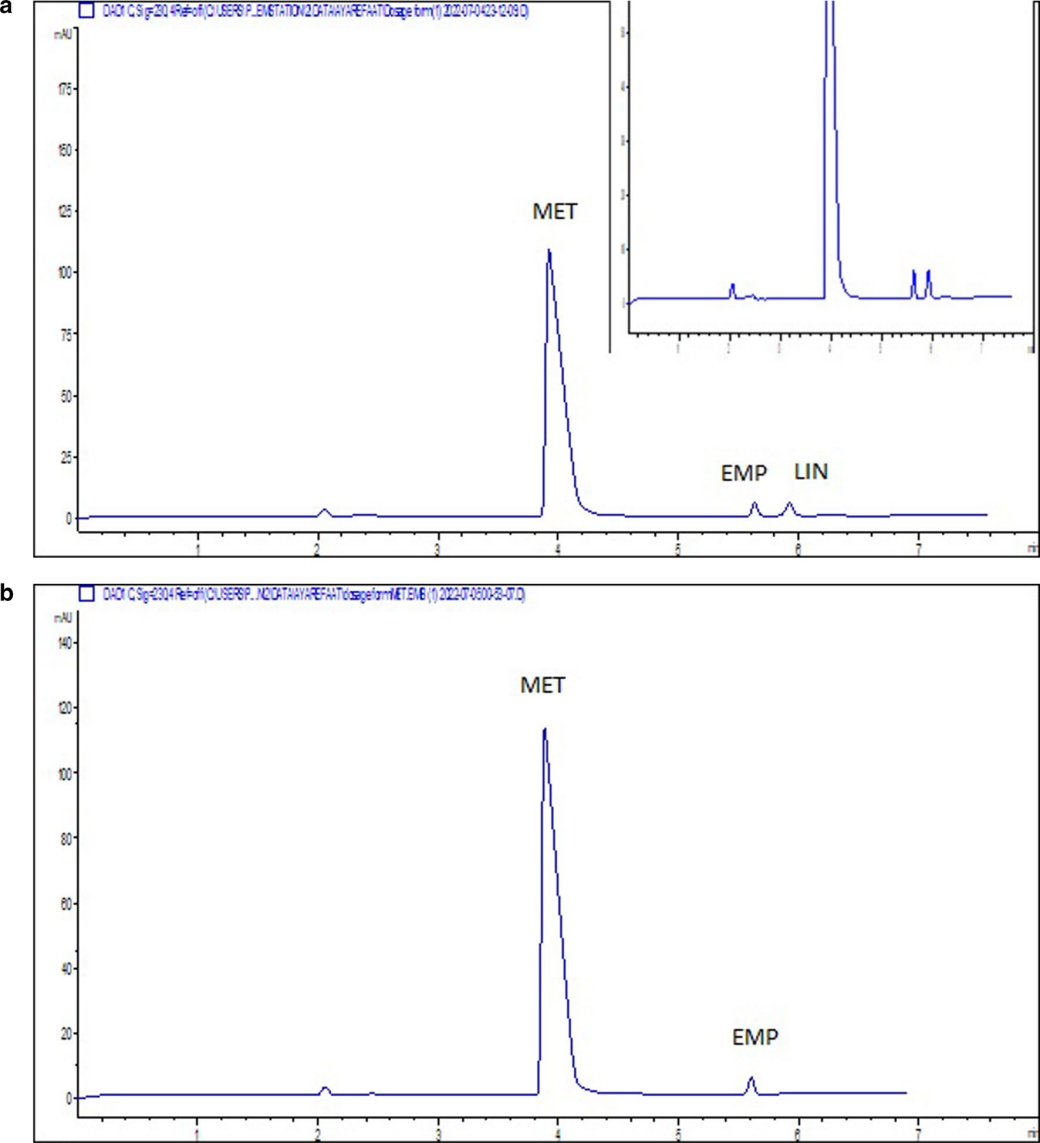

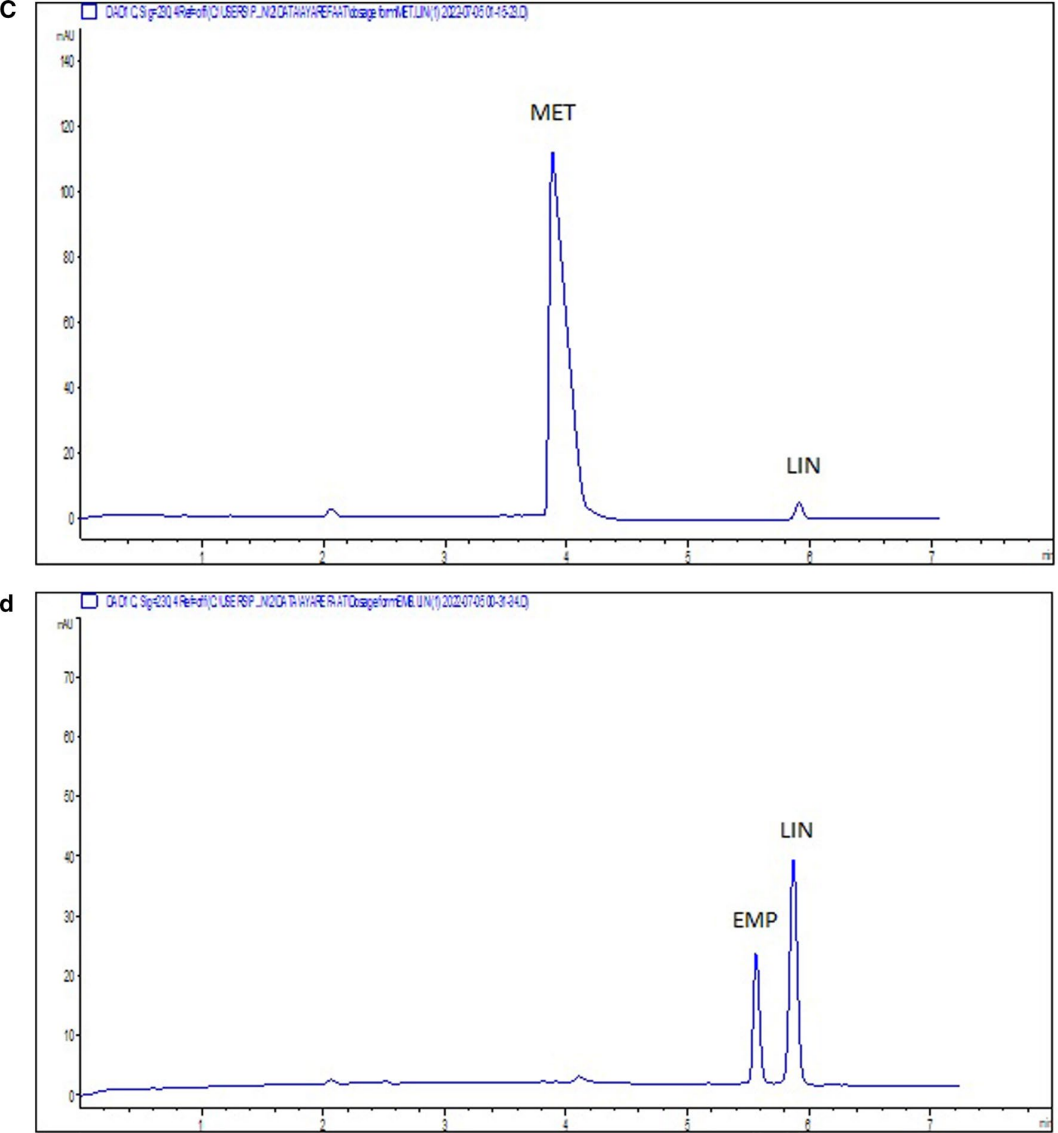
Typical electropherograms of **a** laboratory-prepared tablet solutions of 500 µg. mL^−1^ MET, 12.5 µg. mL^−1^ EMP, 2.5 µg. mL^−1^ LIN,( Mix I) **b** Empagliform® tablets containing 500 µg. mL^−1^ MET and 12.5 µg. mL^−1^ EMP,(Mix II) **c** Gliptalina® tablets containing 500 µg. mL^−1^ MET and 2.5 µg. mL^−1^ LIN,(Mix III) **d** Glyxambi® tablets containing 50 µg. mL^−1^EMP and 25 µg. mL^−1^ LIN ( Mix IV) measured at 230 nm

Lack of tablet existing excipients and additives interference was a confirmation of the specificity of the MEKC method. This was done by the analysis of pharmaceutical dosage forms that represent the cited four mixtures of the three drugs (EMP, LIN and MET). Also, the MEKC method specificity was further confirmed by obtaining acceptable values for the system suitability parameters as EMP, LIN and MET peaks were well separated from each other and showed good resolution values (above 2.25). Moreover, the overlying of the spectra recorded at different wavelength intervals during the registration of each electrophoretic peak in the analysis of dosage forms obtained from the DAD indicates the purity of the peaks (Fig. 3 and Additional file 1: Fig. S4) and the calculated purity angles of EMP, LIN and MET in their purity plots were less than the purity threshold indicating peak purity of the studied drugs.

#### Stability of solutions

Stock solutions and standard solutions of EMP, LIN and MET were found to be stable for one month at 4 ℃ and for 8 h at 25 ℃, respectively. No changes in peak areas and no extra peaks were found.

### Application to pharmaceutical analysis

The applicability of the suggested MEKC method was extended to the analysis of ternary pharmaceutical mixture of Mix I, and three binary mixtures Mix II, Mix III and Mix IV. These prepared tablet solutions ("Analysis of pharmaceutical formulations:" section) representing the quaternary studied mixtures, were analyzed under the optimized MEKC conditions and their electropherograms were shown in Fig. 3. The method was able to determine EMP, LIN and MET with low % error < 1.10, low RSD < 1.95%, and no interference from co-formulated components. Table 2 summarizes the assay results of analysis of the different anti-diabetic mixtures in their different tablet solutions. The results were compared with reported HPLC methods for the assay of Mix I [6] and Mix II [13], CE method for Mix III [22] and spectrophotometric method for Mix IV [21] in their tablets, being taken as the reference methods. The current MEKC mode was compared with other reported methods to check any statistically significant difference between them. Considering the estimated t and F values were < the critical ones which are 2.31 and 6.39, respectively as shown in Table 2. This revealed high degree of agreement between the proposed MEKC method and the reference methods.

**Table 2:** Assay results of MET, EMP, and LIN in their pharmaceutical tablets using the proposed MEKC method

	MEKC Method			Reference Method*		
	MET	EMP	LIN	MET	EMP	LIN
MET/EMP/LIN (Mixture I)^a^ Mean % recovery ± SD RSD % E_r %_ t ^e^ F ^e^	100.27 ± 0.16 0.16 0.27 1.71 1.12	99.84 ± 0.68 0.68 −0.16 0.53 3.04	100.44 ± 0.71 0.71 0.44 2.22 3.91	99.96 ± 0.025 0.025 −0.04	99.65 ± 0.39 0.39 −0.35	99.65 ± 0.36 0.36 −0.35
MET/EMP (Mixture II)^b^ Mean % recovery ± SD RSD % E_r %_ t ^e^ F ^e^	100.04 ± 0.59 0.59 0.04 0.80 5.15	99.60 ± 0.68 0.68 −0.40 2.10 4.00		99.81 ± 0.26 0.26 −0.19	98.88 ± 0.34 0.34 −1.12	
MET/LIN (Mixture III)^c^ Mean % recovery ± SD RSD % E_r %_ t ^e^ F ^e^	100.52 ± 1.50 1.49 0.52 0.12 5.01		100.46 ± 1.96 1.95 0.46 0.07 1.85	100.17 ± 0.67 0.67 0.17		100.54 ± 1.44 1.43 0.54
EMP/LIN (Mixture IV)^d^ Mean % recovery ± SD RSD % E_r %_ t ^e^ F ^e^		99.84 ± 0.80 0.80 −0.16 2.24 1.91	101.10 ± 0.75 0.74 1.10 2.15 3.16		100.83 ± 0.58 0.57 0.83	100.28 ± 0.42 0.42 0.28

*The reference methods used were HPLC methods for Mix I (7) and Mix II (13), CE for Mix III (22) and spectrophotometric method for Mix IV (21)

^a^Labeled to contain 12.5 EMP, 2.5 LIN and 500 METµg.mL^−1^

^b^Labeled to contain 12.5 EMP and 500 MET µg. mL^−1^

^c^Labeled to contain 2.5 LIN and 500 MET µg. mL^−^

^d^Labeled to contain 25 LIN and 50 EMP µg. mL^−^

^e^Theoretical values at 95% confidence level (n = 5) for t and F are 2.31 and 6.39, respectively

## Assessment of greenness and whiteness of the proposed method

The analytical methodology should strike a compromise between being environmentally friendly and functionality in terms of completing the scope of application with a high degree of accuracy and precision, saving time and money, and consuming less waste and harmful chemicals. Consequently, the greenness and whiteness of the current work were assessed and compared with reported HPLC [6], HPTLC [6], UPLC [10] and spectrophotometric [11] methods by using two green approaches, namely, the Analytical Eco-Scale [38] and the most novel Analytical Greenness metric (AGREE) [39] side by side with whiteness appraisal using the multicriteria RGB 12 model [40, 41].

Using the semi quantitative analytical Eco-scale, which depends on subtracting the penalty points (PP) out of total score 100, our proposed MEKC method had a score equal to 86 more than 75, so it is considered an excellent green analysis (Additional file 1: Table S4).

**Fig. 4:**
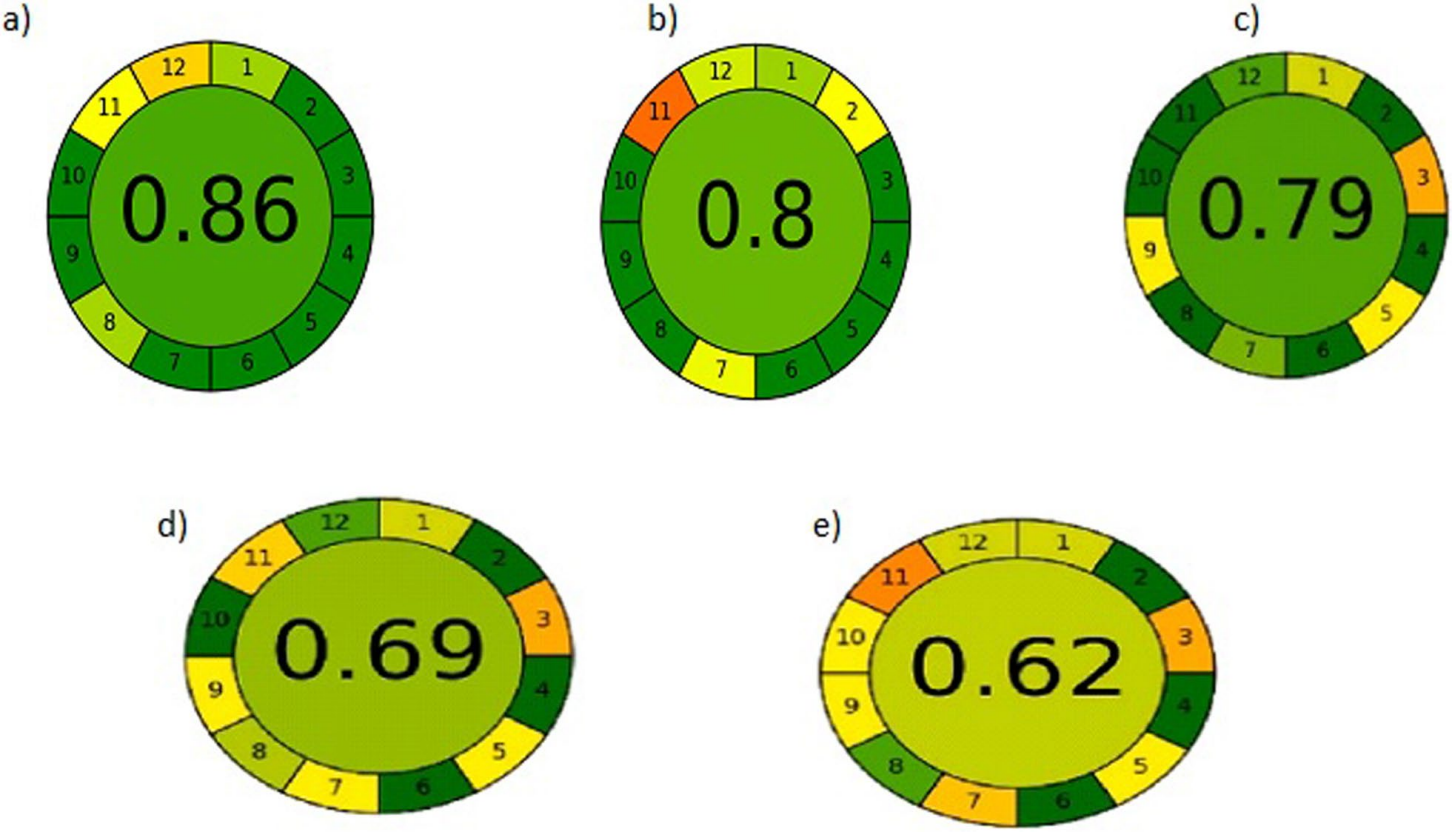
AGREE assessment of the green profile of the evaluated procedures for determination of the three antidiabetic drugs by the proposed MEKC method (**a**), with reported spectrophotometric method, (**b**), reported HPTLC (**c**), reported UPLC (**d**) and reported RP-HPLC method (**e**)

Moreover, using the rapid and easy to apply AGREE tool [38], the proposed MEKC method is the perfect green showing a pictogram with a score 0.86 followed by the spectrophotometric method and finally the chromatographic methods (Fig. 4).

**Fig. 5:**
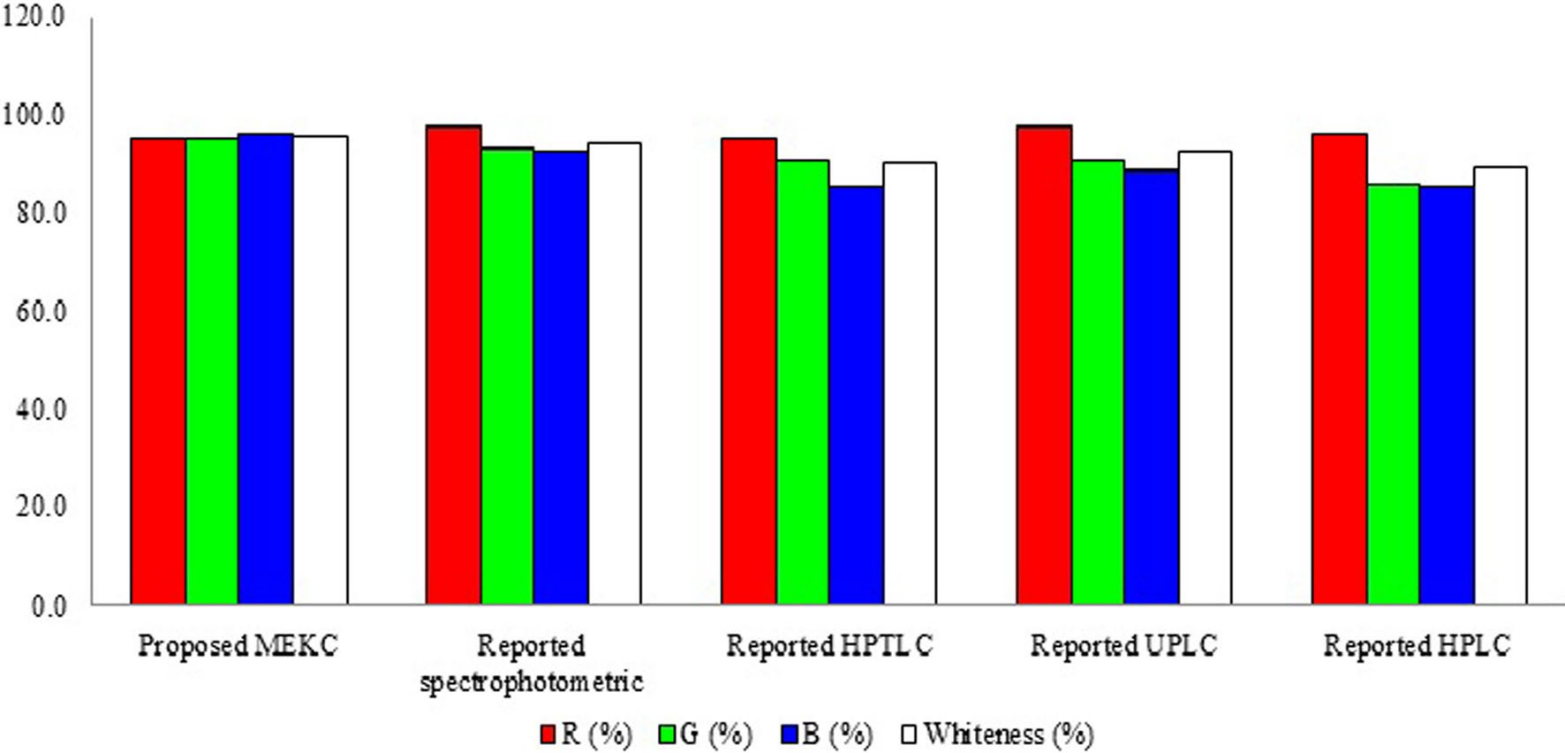
Comparison of the main evaluation outcomes obtained from RGB12 analysis for the proposed MEKC method with the reported methods. The white bar (whiteness %) indicates the arithmetic mean of the three other bars (red, green, and blue)

The most current published multicriteria evaluation technique is the Red Green Blue (RGB) 12 model [40]. Up to 10 analytical approaches can be ranked and compared using the same Excel worksheet. The main evaluation outcomes obtained from RGB12 analysis for the proposed MEKC mode when compared to other reported method are shown in (Fig. 5 and Table 3).

**Table 3:** RGB12 profiles of the proposed MEKC method and other reported methods

Method number	Method name	R (%)	G (%)	B (%)	Whiteness (%)
1	Proposed MEKC	95.0	95.0	96.3	95.4
2	Reported spectrophotometric	97.5	92.9	92.5	94.3
3	Reported HPTLC	95.0	90.4	85.0	90.1
4	Reported UPLC	97.5	90.8	88.8	92.4
5	Reported HPLC	96.3	85.8	85.0	89.0

Whiteness % indicates the arithmetic mean of the three others % (red, green and blue)

To sum up, upon applying the three approaches we found that our proposed MEKC method is superior to reported chromatographic and spectrophotometric methods concerning high separation efficiency with short analysis time, small quantities of reagents and solvents with minimal negative impact on the environment and human health. Thus, it was valuable to develop a MEKC method for rapid, green, white, and effective determination of the three antidiabetic medications.

## Conclusion

A simple, rapid, inexpensive green and white MEKC method has been developed for the first time for the simultaneous determination of the combo pill triple therapy containing LIN and EMP in their lowest critical concentration ratios with MET. To our knowledge, this triple combination and EMP binary combination with MET or LIN were not previously determined using MEKC or even the conventional CE method. The MEKC method for the analysis of MET, EMP and LIN was totally optimized and validated as per the ICH guidelines. Moreover, greenness and whiteness appraisal for the suggested MEKC method were done using two different green metrics (AGREE and Analytical Eco-Scale) and white model (RGB algorithm) ascertaining that our MEKC method was greener and whiter when compared to other published methods.

The proposed MEKC method is characterized by high resolving power that allowed this mode to rapidly analyze in relatively short periods of time a large number of potential drugs with good resolution in the same run despite the large difference between the doses of LIN or EMP with MET. Moreover, minimal sample and aqueous buffer consumption adds the advantages of method’s greenness and economic. This promotes our proposed MEKC method to be used in quality control laboratories not only for its high-through-put but also because it is efficient, simple green and white analytical tool.

## Supplementary Information

**Additional file 1:** Instrumental parameters optimization. **Figure S1**. Effect of pH of 20 mM Tris buffer on the asymmetry factor of EMP (**a**), electropherogram showing EMP shouldered peak obtained upon trying BGE at pH 9 as representative example (**b**). **Figure S2**. Effect of (**a**) SDS concentration (**b**) percent of organic modifier (**c**) applied voltage on the migration time of MET, EMP, and LIN. **Figure S3**. The absorption spectra of (**a**) MET (**b**) EMP and (**c**) LIN of the standard mixture extracted from DAD. **Figure S4**. The peaks purity plot and profile from tablet extract of the three compounds MET, EMP and LIN respectively measured at 230 nm for the analysis of MET/LIN/EMP (Mix I) as a representative example. **Table S1**. System suitability parameters for the MEKC determination of MET, EMP and LIN ternary mixture. **Table S2**. Intra-day and Inter-day precision and accuracy for the determination of MET, EMP and LIN using the proposed MEKC method. **Table S3**. Evaluation of the robustness of the proposed MEKC method for the determination of MET, EMP and LIN ternary mixture. **Table S4**. Analytical eco-scale for assessment of greenness of the proposed MEKC method.

## Data Availability

The dataset supporting the conclusions of this article are included within the article and its additional file.

## Abbreviations

AGREE: Analytical Greenness metric
EMP: Empagliflozin
LIN: Linagliptin
MET: Metformin
t_m_: Migration time
PP: Penalty points
RGB: Red, green, blue
T2DM: Type 2 diabetes mellitus
