# Supplementary material for: Green and white MEKC for determination of different anti-diabetic binary mixtures and their triple-combo pill
Source: BMC Chem. 2023 Jul 25;17(1):86. doi: 10.1186/s13065-023-00997-0 (PMC10367405; doi:10.1186/s13065-023-00997-0)
Supplement: Supplementary file 1 — Additional file 1: Instrumental parameters optimization. Figure S1. Effect of pH of 20 mM Tris buffer on the asymmetry factor of EMP (a), electropherogram showing EMP shouldered peak obtained upon trying BGE at pH 9 as representative example (b). Figure S2. Effect of (a) SDS concentration (b) percent of organic modifier (c) applied voltage on the migration time of MET, EMP, and LIN. Figure S3. The absorption spectra of (a) MET (b) EMP and (c) LIN of the standard mixture extracted from DAD. Figure S4. The peaks purity plot and profile from tablet extract of the three compounds MET, EMP and LIN respectively measured at 230 nm for the analysis of MET/LIN/EMP (Mix I) as a representative example. Table S1. System suitability parameters for the MEKC determination of MET, EMP and LIN ternary mixture. Table S2. Intra-day and Inter-day precision and accuracy for the determination of MET, EMP and LIN using the proposed MEKC method. Table S3. Evaluation of the robustness of the proposed MEKC method for the determination of MET, EMP and LIN ternary mixture. Table S4. Analytical eco-scale for assessment of greenness of the proposed MEKC method. [file 13065_2023_997_MOESM1_ESM.docx]

**Instrumental parameters optimization:**

Different voltages of 20, 25 and 30 kV were applied using the optimized BGE to test for its effect on migration time, resolution, and peak shape. Fig. S2c revealed that by decreasing voltage, migration times of the studied anti-diabetic compounds increased as expected due to the decrease in the EOF. Despite of increasing migration times of the three compounds, resolution was unaffected significantly but MET peak became broad at 20 KV. Therefore, a voltage of 30 kV was selected. As discussed in buffer selection (section 3.1 in text), the Tris buffer was selected not only for EMP peak enhancement but also for minimum Joule heating generation (20 µA). The use of TRIS buffer enables us to use maximum separation voltage 30 KV with low Joule heating generation.

EMP, LIN and MET were analyzed under different values of the applied pressure (40, 50, and 60 mbar) and no significant change was noticed on the migration time of any of MET, EMP and LIN. However, Pressure less than 50 mbar yielded low response, while 50 and 60 mbar no significant change was observed in peak response so 50 mbar was chosen as optimum. Sample injection time was tested in different range from 5–25 s. An injection time less than 10 s yielded low peak responses and above 15 s resulted in broad peaks lead to overlapping between EMP and LIN peaks. Thus, 15 s was selected as optimum regarding EMP, LIN and MET peaks shape, responses, and their separation.

The increase in temperature has an inverse effect on the BGE viscosity with an expected increase in the migration rate (less run time). The separation of standard mixtures of EMP, LIN and MET were investigated at different temperatures 25 ºC ± 5 ºC upon which at 30 ºC MET peak was distorted, so 25 ºC was selected as optimum regarding peak shape, response, and resolution.

a)

**b)**

EOF

EMP

Sup. Figure 1 : Effect of pH of 20 mM Tris buffer on the asymmetry factor of EMP (a), electropherogram showing EMP shouldered peak obtained upon trying BGE at pH 9 as representative example (b).

1. b)

c)

**Sup. Figure 2:** Effect of a) SDS concentration b) percent of organic modifier c) applied voltage on the migration time of MET, EMP, and LIN.

**a) b)**

EMP

MET

**c)**

LIN

**Sup. Figure 3:** The absorption spectra of a) MET b) EMP and c) LIN of the standard mixture extracted from DAD.

MET

EMP

LIN

**Sup. Figure 4** : The peaks purity plot and profile from tablet extract of the three compounds MET, EMP and LIN respectively measured at 230 nm for the analysis of MET/LIN/EMP (Mix I) as a representative example.

**Sup. Table 1: System suitability parameters for the MEKC determination of MET, EMP and LIN ternary mixture.**

| **Parameters** | **MET** | **EMP** | **LIN** |
| --- | --- | --- | --- |
| **t_m_± SD (minutes)** | 3.9±0.04 | 5.5±0.02 | 5.9±0.04 |
| **Capacity factor (k')** | 2.9 | 4.5 | 4.9 |
| **Theoretical plates (N)** | 14857 | 32799 | 29386 |
| **Selectivity (α)** | 1.55^a^ | | 1.09^b^ |
| **Resolution (R_s_)** | 11.78^a^ | | 2.25^b^ |
| **Asymmetry factor (A_f_)** | 0.80 | 0.98 | 0.91 |

System suitability recommendations: k' >2, N > 2000, α > 1, R_s_ > 2 and A_f_ (0.8-1.2)

^a^ Selectivity and resolution values calculated between the two successive peaks of MET and EMP

^b^ Selectivity and resolution values calculated between the two successive peaks of EMP and LIN

Sup. Table 2: Intra-day and Inter-day precision and accuracy for the determination of MET, EMP and LIN using the proposed MEKC method.

| **Concentration**  **(**µg. mL^-1^**)** | | | | Recovery^a^ | | | | | **RSD (%)^b^** | | | | | **E_r_ (%)^c^** | | | |
| --- | --- | --- | --- | --- | --- | --- | --- | --- | --- | --- | --- | --- | --- | --- | --- | --- | --- |
| MET | | EMP | LIN | MET | EMP | | LIN | | MET | | EMP | | LIN | MET | EMP | | LIN |
| 1. Intra-day Precision and Accuracy (n=3) | | | | | | | | | | | | | | | | | |
| 500 | 12.5 | | 2.5 | 99.54 | 98.27 | | 99.77 | | 1.74 | | 1.66 | | 1.65 | -0.46 | -1.73 | | -0.23 |
| 10 | 100 | | 100 | 100.48 | 98.20 | | 98.76 | | 1.98 | | 1.23 | | 0.25 | 0.48 | -1.80 | | -1.24 |
| 200 | 50 | | 25 | 101.94 | 98.97 | | 99.78 | | 0.47 | | 0.39 | | 1.84 | 1.94 | -1.03 | | -0.21 |
| 1. Inter-day precision and accuracy (n=9) | | | | | | | | | | | | | | | | | |
| 500 | 12.5 | | 2.5 | 101.91 | | 101.59 | | 98.75 | 0.79 | 0.62 | | 0.91 | | 1.91 | | 1.59 | -1.25 |
| 10 | 100 | | 100 | 99.97 | | 100.49 | | 101.90 | 0.57 | 0.42 | | 1.78 | | -0.03 | | 0.49 | 1.90 |
| 200 | 50 | | 25 | 98.03 | | 98.77 | | 101.02 | 1.96 | 0.66 | | 0.93 | | -1.97 | | -1.23 | 1.02 |

**^a^** Mean recovery for three determinations**.**

**^b^** % Relative standard deviation.

**^c^** % Relative error.

**Sup. Table 3 :** **Evaluation of the robustness of the proposed MEKC method for the determination of MET, EMP and LIN ternary mixture.**

| **Parameters** | **MET** | | **EMP** | | **LIN** | |
| --- | --- | --- | --- | --- | --- | --- |
|  | **RSD%**  **of peak areas** | **t_m_ ± SD** | **RSD%**  **of peak areas** | **t_m_ ± SD** | **RSD%**  **of peak areas** | **t_m_ ± SD** |
| 1. **Buffer concentration(** 20 mM ± 2 mM) | 1.62 | 3.90±0.09 | 0.74 | 5.51±0.01 | 0.53 | 5.90±0.06 |
| 1. **SDS concentration**   (50 mM ± 2 mM) | 0.36 | 3.90±0.05 | 1.31 | 5.49±0.21 | 0.87 | 5.94±0.07 |
| 1. **pH of buffer**   **(10 ± 0.2 pH units)** | 0.64 | 3.95±0.01 | 0.58 | 5.50±0.09 | 0.94 | 5.91±0.09 |
| 1. **Percent of organic modifier (**10 ± 1%) | 0.58 | 3.98±0.08 | 0.49 | 5.50±0.12 | 0.45 | 5.93±0.20 |
| 1. **Wavelength**   **(230 ± 2 nm)** | 1.09 |  | 0.82 |  | 0.86 |  |

**Sup. Table 4: Analytical eco-scale for assessment of greenness of the proposed MEKC method.**

| **The penalty points (PPs) to calculate** **Analytical Eco -scale** | | | |
| --- | --- | --- | --- |
| **Reagent** | **Number of pictograms** | **Signal word** | **Penalty points** |
| Methanol (10% v/v) | 3 | Danger | 6 |
| Sodium Dodecyl sulphate (SDS) | 3 | Danger | 6 |
| Tris buffer | 1 | Warning | 1 |
| **Total penalty points for reagents**  N.B: Reagent amount used < 10 mL (g) | | | **13** |
| **Instrument** |  | | |
| Energy | <0.1 KWH per sample | | 0 |
| Waste | < 1 mL | | 1 |
| Occupational hazards | Analytical process hermitization | | 0 |
| **Total penalty points (sum of instrument points)** | | | **1** |
| **Final total penalty points (sum of reagent and instrument points)** | | | **14** |
| **Analytical eco-scale score** | | | **86** |
